# Sheer Time Spent Expecting or Maintaining a Representation Facilitates Subsequent Retrieval during Sentence Processing

**Published:** 2020

**Authors:** Hossein Karimi, Michele Diaz, Eva Wittenberg

**Affiliations:** Department of Psychology, 109 Moore Building, University Park, PA 16801 USA; Department of Psychology, 356 Moore Building, University Park, PA 16801 USA; Department of Linguistics, 9500 Gilman Drive, La Jolla, CA 92093 USA

**Keywords:** semantic complexity, time spent, attention, encoding, retrieval

## Abstract

Previous research has shown that modified noun phrases (henceforth NPs) are subsequently retrieved faster than unmodified NPs. This effect is often called the “semantic complexity effect”. However, little is known about its mechanisms and underlying factors. In this study, we tested whether this effect is truly caused by the semantic information added by the modification, or whether it can be explained by the sheer amount of time that the processor spends expecting or maintaining an NP in the encoding phase. The results showed that time spent expecting or maintaining an NP can explain the effect over and above semantic and/or syntactic complexity. Our results challenge the current memory-based mechanisms for the modification effect such as the “distinctiveness” and “head-reactivation” accounts, and offer new and valuable insight into the memory processes during sentence comprehension.

## Background

Language processing necessarily depends on encoding, storage and retrieval of information. For example, successful resolution of long-distance syntactic dependencies such as (1), and referential dependencies such as (2), depend on the successful retrieval of words from earlier parts of the sentence:

1. It was the bear that the hunters chased in the cold forest yesterday.
2. The bear fell into a trap when it was running from the hunters.

Specifically, successful processing of “chased” in (1) and “it” in (2) depends on successful retrieval of “bear”. In (1), “bear” has been moved from the position immediately following “chased” to an earlier position to create emphasis via a syntactic operation called “clefting”. When “chased” is being processed, the displaced word (i.e., *bear*) needs to be retrieved as its syntactic object (e.g., Kluender & Kutas, 1993; McElree, 2000; Tanenhaus et al., 1985). In (2), there is a referential dependency between the pronoun “it” and “bear” such that “it” assumes meaning by referring to “bear”. As such, successful processing of the pronoun is contingent on retrieval of its referent (i.e., *bear*; Dell et al., 1983; Gernsbacher, 1989; Lucas et al., 1990; MacDonald & MacWhinney, 1990).

An important question in psycholinguistics is which factors influence the retrieval difficulty of previously encoded NPs. Recent years have seen numerous studies showing that for both syntactic and referential dependencies, enriching an NP through modification at encoding (e.g., *the injured and dangerous bear*) facilitates its subsequent retrieval compared to leaving the same NP unmodified (i.e., *the bear*; e.g., Hofmeister, 2011; Hofmeister & Vasishth, 2014; Karimi et al., 2014, 2018; 2019; Karimi & Ferreira, 2016; Troyer et al., 2016). In a seminal study, Hofmeister (2011) showed that pre-modified noun phrases such as *the alleged Venezuelan communist* result in faster reading times at a subsequent verb that triggers the retrieval of that NP compared to unmodified NPs such as *the communist*. Importantly, it does not matter whether the modifying information is added pre-nominally (i.e., *the injured and dangerous bear*) or post-nominally (i.e., *the bear that was injured and dangerous*, Karimi et al., 2019). In fact, Karimi & Ferreira (2016) showed that ambiguous pronouns tend to be interpreted as referring to post-modified rather than unmodified NPs, suggesting that retrieval is easier for post-modified than unmodified NPs.

However, multiple possible mechanisms could explain these results. We consider two competing accounts:

1. *Complexity account*: The added semantic content and the greater syntactic complexity of modified NPs result in more robust encoding through a detailed semantic representation of the NP, and therefore a facilitated subsequent retrieval (Hofmeister, 2011). Thus, the source of the easier retrieval lies within the domain of conceptual construal.

2. *Time-dependent attention account*: The processer necessarily spends more time attending to the head noun when it is modified, compared to when it is unmodified. This leads to more robust encoding and subsequent retrieval facilitation through increased attentional resources. In this account, the source of easier retrieval stems from time and attention spent on the noun during encoding, not from detailed semantic complexity. This account should hold for both post-and pre-nominally modified NPs: In the case of post-modified NPs (*the bear that was injured and dangerous*), the head noun (*bear*) is encountered before the modifying information (*injured and dangerous*), and as such, the processor necessarily spends more time maintaining the representation of the head noun when there is post-modifying information compared to when there is no such information. In addition to time spent, post-modifying information might also recruit more attentional/memory resources because post-modifiers tend to convey more complex information (Karimi et al., 2019; also see Dillon et al., 2017).

In the case of pre-modified NPs (*the injured and dangerous bear*), pre-modifying information is encountered before the head noun. However, because the determiner (*the*) predicts an upcoming head noun, the processor spends more time *expecting* the head noun in the case of pre-modified relative to unmodified NPs. Similar to post-modifiers, longer time spent expecting the head noun in the case of premodifiers might result in heightened attention, leading to more robust encoding of the head noun when it is encountered, which then facilitates its subsequent retrieval.

We disentangle the complexity account from the time-dependent attention account in three experiments. In Experiments 1 and 2, we tested the retrieval of pre-modified and post-modified NPs, respectively, during the processing of syntactic dependencies as in (1). In Experiment 3, we examined the retrieval of post-modified NPs during referential dependency processing as in (2).

## Experiment 1

We created 60 experimental sentences such as (3). However, three items were later removed due to coding errors. Participants read the sentences for comprehension in a self-paced reading paradigm. Two versions of each experimental sentence contained Unmodified (e.g., *the bear*) and Pre-modified (e.g., *the injured and dangerous bear*) NPs such as (3a) and (3b), respectively, to try to replicate previous findings on the pre-modification effect (Hofmeister, 2011).

(3)

**Table:** 

3a	Unmodified	It was the bear that the hunters chased in the cold forest yesterday.
3b	Pre-modified	It was the injured and dangerous bear that the hunters chased in the cold forest yesterday.
3c	Cue-Giving	It was the 부 상 당 bear that the hunters chased in the cold forest yesterday.
3d	No-Cue	It was 그 부 상 당 bear that the hunters chased in the cold forest yesterday.

We also added two conditions where the modification was replaced with as many Korean characters as there were English words in the Pre-modified condition. This manipulation simulated additional time spent on the NP in the Pre-Modified condition, but without adding syntactic or semantic content.

In order to understand whether prediction of an NP can explain the retrieval effect, in the Cue-Giving condition (3c), the masking Korean characters were presented with English syntactic cues (i.e., the determiner *the*), revealing that the masking characters were modifying an upcoming head noun. In the No-Cue condition, the syntactic cue was replaced with a masking character too, making the function of the masking characters unknown: The sentence could go on with an adjective (*It was interesting…*) or verb participle (*It was decided…*), for instance.

Importantly, the Cue-Giving condition speaks to both the time-dependent attention account as well as syntactic complexity. This is because the syntactic cues reveal the type of syntactic construction associated with the head noun, and therefore maintain its syntactic complexity. The No-Cue condition, on the other hand, only tests the time-dependent attention mechanism, because no syntactic cues are provided and therefore there is no reason to expect and devote attentional/memory resources to an upcoming head noun. Note that by providing a syntactic cue revealing that the head noun is imminent, the Cue-Giving condition also channels attentional resources onto the head noun more efficiently.

### Predictions

If more robust encoding of semantically rich NPs is caused by the additional semantic information and/or syntactic complexity, and time spent has no effect, then the critical verb should be read faster in the Pre-Modified than in the Unmodified condition. However, the critical verbs in the Cue-Giving and No-Cue conditions should be read as fast as those in the Unmodified condition. If time spent expecting the head noun and the concomitant heightened attention, as well as syntactic complexity, matter, then the verbs in the Cue-Giving condition should also be read faster than those in the Unmodified condition. Finally, if the previously reported effects are only due to time spent expecting an upcoming word (namely, any word, not necessarily the head noun), and allocation of attentional resources to it, then the critical verbs in the No-Cue condition should also be read faster than those in the Unmodified condition.

### Stimuli

All experimental stimuli consisted of a clefted subject NP (such as *bear* in 3), and a subsequent clause with a verb whose processing depended on retrieval of the target NP (e.g., *chased* in 3). In addition to the experimental stimuli, we also created 30 fillers, half of which included a random number of masking characters at random parts in the sentence. 16 of the critical sentences and 18 fillers were followed by a comprehension question to encourage participants to pay close attention to the task. The experiment was programmed and run in Ibex Farm (http://spellout.net/ibexfarm/).

### Participants

Sixty participants were recruited from Amazon’s Mechanical Turk (Mturk). We restricted the participants to native speakers of English who resided in the US with HIT approval rates of equal or greater than 95%. Participants also had to indicate whether they spoke any languages other than English. Using this data, we ensured that none of the participants spoke or read Korean. The task took approximately 40 minutes, and each participant was paid $2.

### Procedure

Each trial started with participants viewing dashed lines corresponding to the number of words contained in the current sentence. Then the participants read the sentences by pressing the spacebar on the keyboard, which revealed the words one at a time. As each word appeared, the preceding word disappeared from the screen. If the current item had a comprehension question, it would appear on the screen after the last word of the sentence was read, and the participant had to indicate whether it was true or false by clicking on the TRUE or FALSE words that appeared below the sentence. If there was no question for the current trial, the next trial would automatically start when the participants pressed the space bar. There were two practice trials at the beginning of the experiment so that the participants had a chance to become accustomed to the experimental procedure.

### Statistical analyses

Our analyses closely followed the steps taken by Hofmeister (2011). We first removed unreasonable reaction times (RTs), namely those faster than 100ms and slower than 2500ms. We then removed RTs that fell 2.5 standard deviations below or above the mean RT for each subject, and each sentence region. Next, we conducted a regression model predicting RTs by log-transformed trial number in the experimental session, word length, and the restricted cubic spline of the word position within each sentence, as well as all the possible interactions between these three predictors. We then used the residuals of this model as the critical measure to test the effects of our predictors of interest. As the final analysis step, we ran mixed effects regression models on the residual reading times, always with full random-effects structures (i.e., random intercepts for subjects and items, as well as by-subjects and by-items random slopes for the effect of our predictor, Barr et al., 2013). The predictor was dummy coded such that the Unmodified condition was taken as the baseline and the three remaining conditions were compared to it. We calculated *p* values using the normal approximation method (Barr et al., 2013). We used the same analysis procedure in all experiments (see below). It is important to mention that trial number, word length and word position all had large significant effects on readings times in all experiments.

### Results

**Figure 1.**
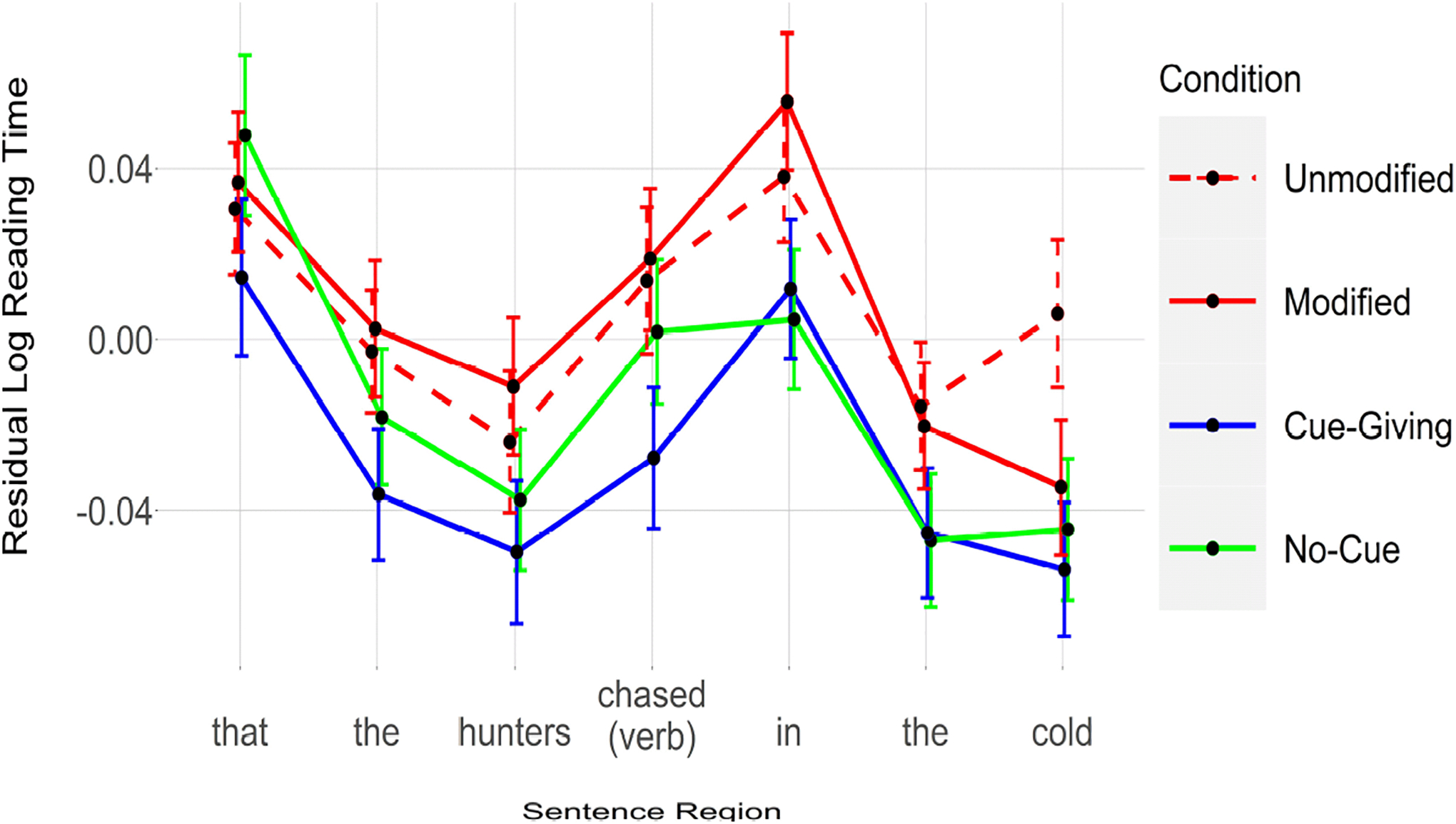
Reading times for each condition, Experiment 1. Error bars represent 95% confidence interval of the mean.

Figure 1 shows reading times for each condition and Table 1 reports the results of our statistical analyses. As can be seen in this table, reading times were faster on the second word preceding the critical verb (i.e., the first *the*) in the Cue-Giving condition compared to the Unmodified condition. The critical verb (i.e., *chased*) was read reliably faster in the Cue-Giving condition than in the Unmodified condition. The two words following the critical verb (i.e., *in* and *the*) were read significantly faster in both the Cue-Giving and No-Cue conditions. Finally, the third word following the verb (i.e., *cold*) was read faster in the all three conditions relative to the Unmodified condition.

### Discussion

We replicated the standard semantic complexity effect, with modified NPs facilitating subsequent reading times (e.g., Hofmeister, 2011; Karimi & Ferreira, 2016; Troyer et al., 2016). However, this effect emerged rather late (i.e., on the third word following the retrieval trigger; but see Experiment 2).

Importantly, our results revealed faster reading times at the retrieval site and the following regions when the English modifications were replaced with masking characters, regardless of whether a determiner predicted an upcoming NP (i.e., in both the Cue-Giving and No-Cue conditions). This pattern of results shows that ease of subsequent retrieval is not necessarily a function of representational complexity in the form of semantic richness and/or syntactic complexity. Rather, the mere amount of time that the processor expects an upcoming noun enhances encoding and therefore facilitates subsequent retrieval.

In fact, the late emergence of the semantic complexity effect (i.e., Modified vs. Unmodified) may suggest that time spent causes more robust encoding than semantic complexity, perhaps because the semantic content consumes some of the attentional resource that would otherwise be devoted to encoding the target NP (also see Experiment 2 below).

Note that, overall, the effect appears later and is relatively weaker in the No-Cue compared to the Cue-Giving condition, suggesting that syntactic cues (i.e., the determiner in our case) strengthen the effect of time spent, perhaps by directing attention to the upcoming head noun more efficiently (perhaps through prediction). Another important observation is that the effect already appeared on the second word preceding the critical word (e.g., the first *the*) in the Cue-Giving condition. Because no retrieval should be triggered on this word, this effect is somewhat surprising. We think this is probably because the integration of all the words following the target NPs (e.g., *bear*) is easier when that word is encoded more robustly (due to time spent). In fact, based on Figure 1, reading times are faster for Cue-Giving and No-Cue conditions relative to the baseline (Unmodified) condition for all the words. This overall speed-up might occur because a more robust representation of the target word (i.e., *bear*) might facilitate the integration of new information in general, regardless of whether the new information triggers retrieval of a specific previously encoded NP or not. Importantly, similar early effects have been reported by previous studies (e.g., Hofmeister & Vasishth, 2014; Karimi et al., 2019).

## Experiment 2

In this experiment, we tested the effect of post-modification on resolving syntactic dependencies. To this end, we used the same stimuli as in Experiment 1, but revised the critical sentences such that the modifications were following the clefted NPs in a relative clause. Again, in the Cue-Giving condition, the modifying words were replaced with masking characters, and in the No-Cue condition, the relative pronoun and the auxiliary (i.e., *that was*) were masked too. Example experimental stimuli are shown in (4). We also increased the number of participants to 116 to maximize power.

(4)

**Table:** 

4a	Unmodified	It was the bear that the hunters chased in the cold forest yesterday.
4b	Post-Modified	It was the bear that was injured and dangerous that the hunters chased in the cold forest yesterday.
4c	Cue-Giving	It was the bear that was 부 상 당 that the hunters chased in the cold forest yesterday.
4d	No-Cue	It was the bear 그 건 부 상 당 that the hunters chased in the cold forest yesterday.

### Results

**Figure 2.**
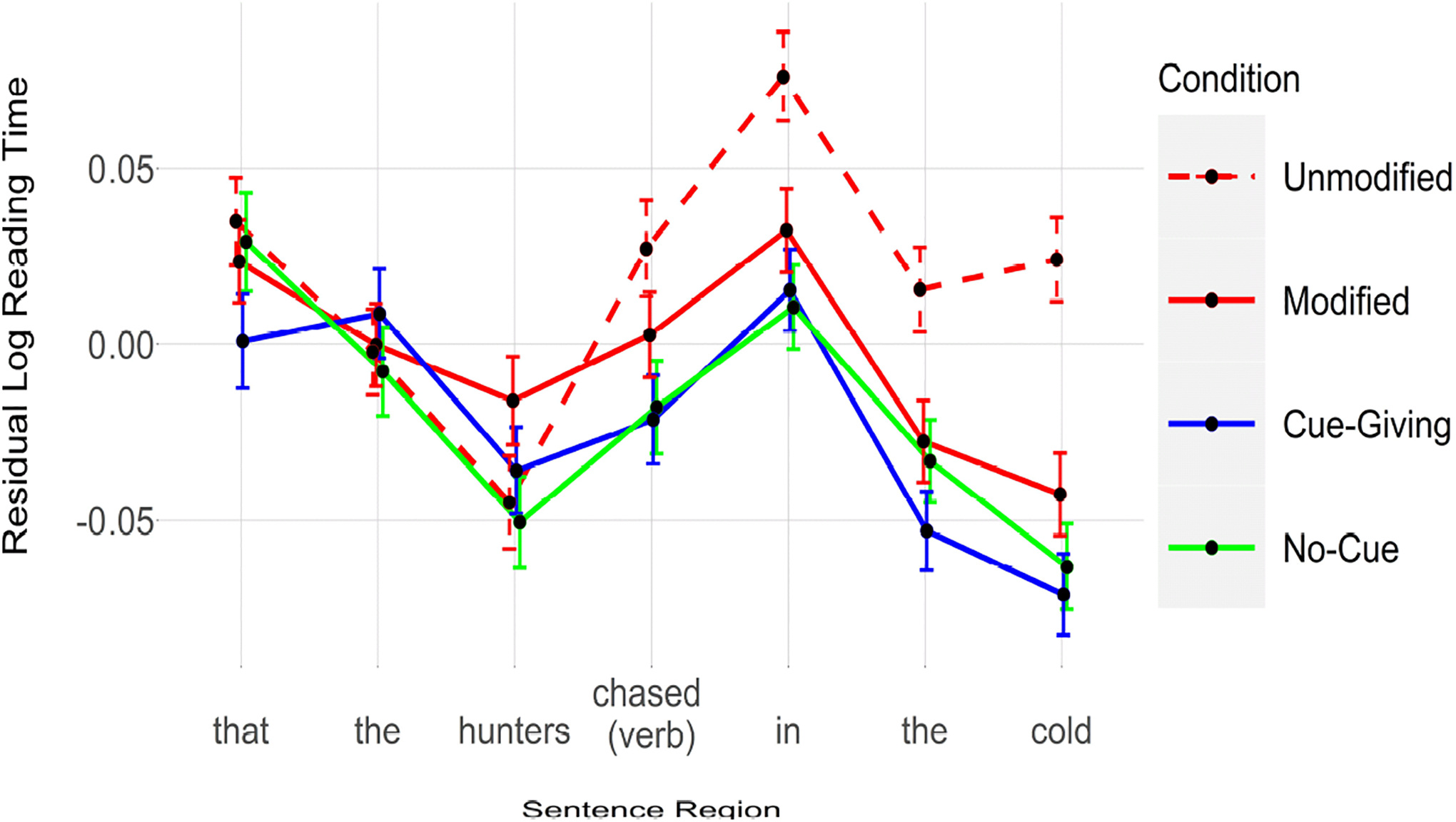
Reading times for each condition. Experiment 2. Error bars represent 95% confidence interval of the mean.

Figure 2 shows reading times for each condition and Table 2 reports the results for Experiment 2. As is clear from this table, the third word preceding the critical word (i.e., *that*) was read faster in the Cue-Giving condition. The word immediately preceding the critical word (i.e., *hunters*) exhibited a reverse complexity effect such that it was read *more slowly* in the Modified than in the Unmodified condition. The critical verb (*chased*) and the three following words (*in the cold*) were all read significantly faster in the Modified, Cue-Giving and No-Cue conditions relative to the Unmodified (baseline) condition.

### Discussion

Again, we replicated the standard semantic complexity effect, with semantically richer and syntactically more complex NPs resulting in facilitated subsequent retrieval (e.g., Hofmeister, 2011; Karimi & Ferreira, 2016; Troyer et al., 2016). Consistent with Experiment 1, the results of Experiment 2 showed that when the processor maintained the memory representation associated with the displaced NP for a longer time, that representation was subsequently retrieved more easily, providing further support for the time-dependent attention account. Based on this hypothesis, the sheer amount of time spent with a representation might lead to heightened attention and therefore a more robust encoding, independent of extra semantic content and/or syntactic complexity (see Introduction). Somewhat surprisingly, we also observed reliably faster reading times for the third word preceding the critical verb in the Cue-Giving condition. We argue that such early effects are perhaps caused by an overall speed-up effect due to robust representations for the target NP (i.e., *bear*; see above). We also observed an unexpected reverse semantic complexity effect on the word immediately preceding the critical word, with slower reading times for the English modified than for the unmodified NP. This effect may be spurious or may reflect a (late) processing cost associated with the temporary ambiguity present in the sentences of this experiment. Specifically, given the clefted structure of sentences, the target noun and the modifying relative clause (*It was the bear that was injured and dangerous*) could be viewed as a complete sentence, making the rest of the sentence surprising for the English modified condition, and perhaps leading to elevated reading times on the next content word *(hunters*).

## Experiment 3

In this experiment, we wished to investigate the effect of time-dependent attention on referential (rather than syntactic) dependences. As mentioned above, pronouns have been shown to trigger the retrieval of their referents (i.e., the NPs they refer to). Thus, pronouns also provide a reasonable testing ground for the effect of time vs. complexity. A sample experimental item is shown in (5). 116 participants took part in this experiment.

(5).

**Table:** 

5a	Unmodified	The bear fell into a trap when it was running from the hunters.
5b	Post-Modified	The bear that was injured and dangerous fell into a trap when it was running from the hunters.
5c	Cue-Giving	The bear that was 부 상 당 fell into a trap when it was running from the hunters.
5d	No-Cue	The bear 그 건 부 상 당 fell into a trap when it was running from the hunters.

### Results

**Figure 3.**
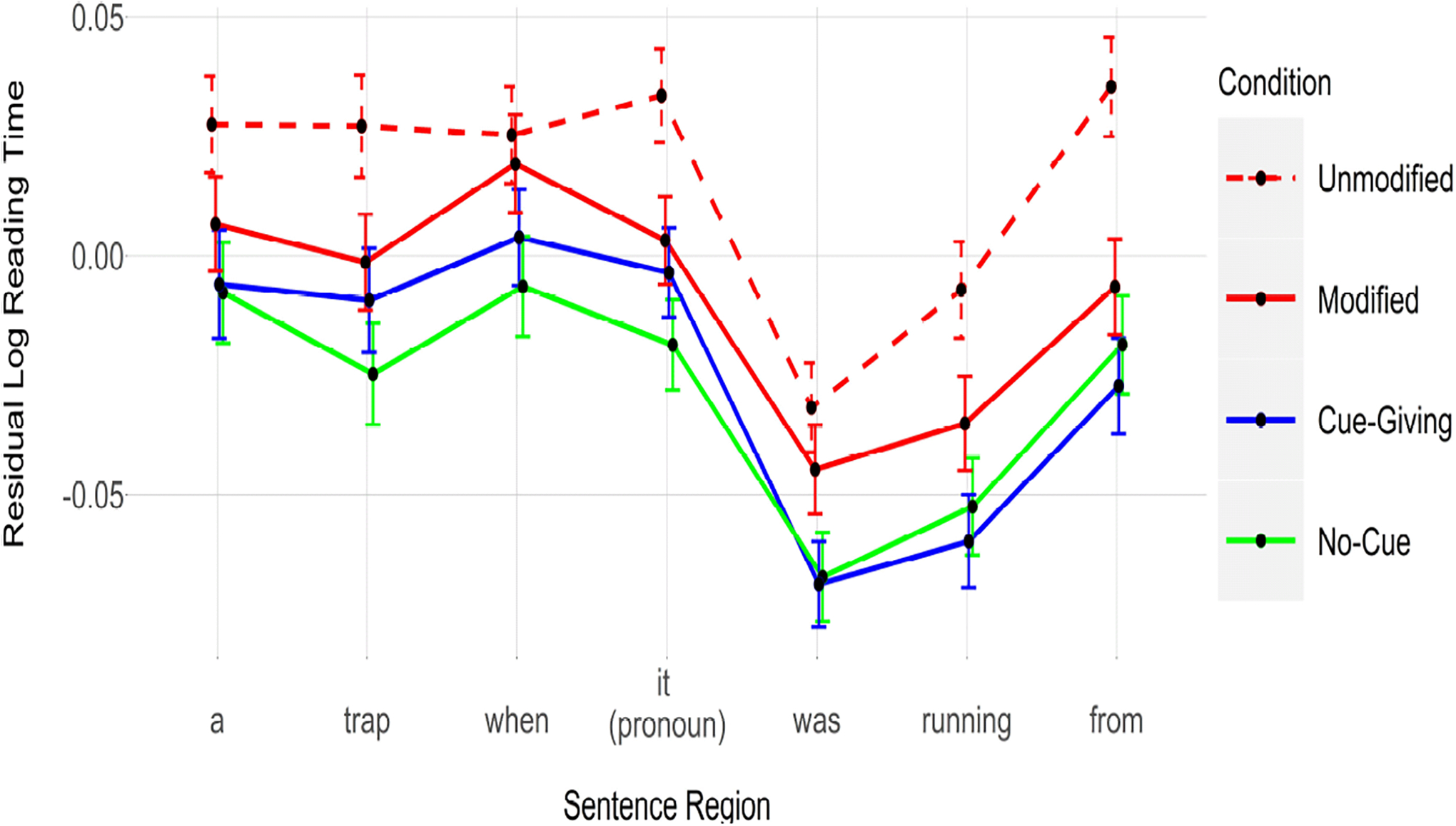
Reading times for each condition. Experiment 3. Error bars represent 95% confidence interval of the mean.

Figure 3 shows the reading time for each condition and Table 3 reports the results of our statistical analyses. As is clear from Table 3, with the exception of the two words immediately preceding and following the critical pronoun (i.e., *when* and *was*) in the Modified condition, all the words were significantly faster in the Modified, Cue-Giving and No-Cue conditions relative to the Unmodified condition.

### Discussion

Unlike Experiments 1 and 2, faster reading times were observed for almost all regions in this experiment. There are substantial differences in the structure of the sentences in this experiment relative to the previous two experiments. Specifically, the sentences in this experiment were not cleft constructions and did not include a second character (*hunters*). Moreover, and as mentioned above, we think these early effects likely reflect a general speed-up due to ease of integration of words with the target when it is encoded more robustly and therefore is more activated in memory. In Experiment 3, this general integration ease might have been enhanced because the verb of the main clause (*fell*) may already reactivate the target NP’s representation, giving it an extra activation boost before the pronoun is encountered. Critically, even without regard to what is causing these early effects, these effects are emerging for all three conditions of interest, indicating that whatever it is that semantic complexity does, time-dependent attention does too.

Thus, consistent with the results of Experiments 1 and 2, Experiment 3 also showed that when a target NP was followed by masking characters, giving the processor a longer time to maintain the associated representation, the retrieval of that representation was easier at a later point. This pattern of results clearly shows that it is not so much the semantic content and/or the syntactic complexity of modified NPs that facilities subsequent retrieval. Rather, it is the time the processer spends with a representation and the resulting heightened attention allocated to that representation.

## General Discussion

In three self-paced reading experiments, we asked whether the retrieval effects on modified NPs reported in the literature (Hofmeister, 2011; Hofmeister & Vasishth, 2014; Karimi et al., 2014, 2018; 2019; Karimi & Ferreira, 2016; Troyer et al., 2016) are due to the added semantic content and/or the greater complexity of modified NPs, or, alternatively, due to the longer time that the processor spends with or expects the target representation, and the concomitant heightened attention to the target NP’s representation.

We examined the retrieval of pre-modified and post-modified NPs during syntactic dependency resolution (Experiments 1 and 2, respectively), and referential dependency resolution (Experiment 3). The critical NPs were either unmodified or modified, allowing us to test previously reported results. To introduce the “time” element, we replaced English modifying words with Korean masking characters, and ensured that participants did not know any Korean. In addition to replicating the modification effect, all three experiments clearly showed that maintaining or expecting the target NP for a longer time essentially produces the same retrieval benefit as semantic modification does, suggesting that the underlying mechanism for the modification effect is likely time and the concomitant increased attention to the associated NP.

For instance, as mentioned in the Introduction, in the case of pre-modifiers, the head (critical) noun is revealed after the masking characters, and, consequently, the processor is forced to expect an upcoming word (either the head noun or any other word) for a longer time, which might increase attention. In the case of post-modifiers, the head noun is given and the masking characters follow it. As such, the processor spends more time maintaining the head noun’s representation in memory. Interestingly, post-modifiers have also been argued to increase attention because they tend to convey more complex information (Karimi et al., 2019). Thus, we argued that time spent expecting or maintaining an NP should result in enhanced encoding and therefore facilitated subsequent retrieval.

An important aspect of our results is that English syntactic cues, namely the determiners (*the*) in Experiment 1, and the relative pronouns and the following auxiliaries in Experiments 2 and 3, did not modulate the time/attention effect (although they did result in relatively stronger and earlier effects). The logic here was that when syntactic cues are present, they constrain the masking characters’ function to modifying the target NP (the Cue-Giving condition). In the absence of such cues, however, the masking characters could mask any syntactic category (the No-Cue condition). So, if syntactic complexity played a role producing the modification effect, we should have observed facilitated retrieval when syntactic cues were present, but not when they were absent. However, no difference was observed as a function of the presence of syntactic cues, suggesting that sheer time spent rather than syntactic complexity is the key factor for the modification effect. Note that the overall stronger effects for the Cue-Giving relative to the No-Cue condition could be caused by the fact that syntactic cues channel attentional resources onto the head noun more efficiently.

One tempting alternative explanation for our results is lower processing demands associated with having to integrate no (or little) extra information with the head noun when English modifying words were replaced with masking characters. However, note that low processing cost also applies to the Unmodified condition, but this condition consistently produced the longest reading times across all three experiments. Thus, less processing effort is unlikely to have caused our results (also see Hofmeister, 2011).

Our results have important implications for cue-based retrieval theories of language comprehension (Jäger et al., 2017; Lewis et al., 2006; Lewis & Vasishth, 2005). Specifically, these theories offer two potential explanations for the modification effect: Under the *distinctiveness* account, due to the added semantic information contained in modifications, modified NPs result in representations that are more distinct from other representations. As such, the retrieval operation suffers less from other interfering representations, leading to easier retrieval. Under the *head-reactivation* account, information whose processing depends on a particular word causes reactivation of that word in memory. Translated to the modification effect, this means that processing modifying words causes the head noun to be re-activated in memory, leading to higher levels of ultimate activation for the head noun when it is modified than not (Hofmeister, 2011).

Our results call both the distinctiveness and the head-reactivation accounts into question. The masking characters did not add any information to the representation of the head noun; thus, the distinctiveness account cannot explain our results. Similarly, because the masking characters necessarily could not be integrated with the head noun, and because head-reactivation is argued to depend on integration, the need to reactivate the head noun was obviated for conditions involving masking characters. This was even more relevant for the No-Cue than for the Cue-Giving condition, because the determiner constrained the linguistic function of the masking characters in the Cue-Giving condition, and thus might have caused some head-reactivation through syntactic integration of the masking characters. However, the absence of a constraining article in the No-Cue condition makes integration and therefore head-reactivation highly unlikely. Thus, we argue that the time spent maintaining or expecting a representation and the concomitant heightened attention are the key factors underlying the modification effect. Our account cannot distinguish between the possibility that it is attention alone, more time alone, or a combination of both, that contributes to easier retrieval; but it is now evident that one or both of these factors plays key a role. We hope to investigate the relative effects of sheer time vs. heightened attention in future research.

Two aspects about the design of our experiments might challenge our conclusions. First, in the Cue-Giving condition of Experiment 1, it is not clear why the masking characters should necessarily be interpreted as modifying an upcoming head noun. In fact, the first masking character could potentially be the head noun, rendering function of the rest of the characters unknown. We argue this is a remote possibility because the article *the* is very strongly associated with a following noun in English. Thus, given that human sentence processing system prefers parsimony over complexity, namely, building the simplest syntactic structure possible (Frazier & Rayner, 1982), interpreting the immediately following masking character as the head noun would mean that the remaining masking characters should have assumed different syntactic classes (e.g., a verb), complicating the unfolding syntactic structure of the sentence. However, anticipating a head noun would render the current syntactic structure simpler. Moreover, our results from Experiment 1 actually show the strongest effect for the Cue-Giving condition, which is consistent with the idea that people *did* anticipate a head noun, and therefore channeled their attention to it. Second, in the Cue-Giving and No-Cue conditions of Experiments 2 and 3, it may not be clear why the participants should spend the time on the masking characters maintaining the head noun in memory rather than trying to make sense of the masking characters themselves. Again, we argue that although not impossible, this is an unlikely scenario because the head noun is (most likely) the most accessible item in memory at the time the masking characters are being read. As such, between maintaining the already-active head noun and trying to make sense of unfamiliar and, to the current participants, meaningless characters, maintaining a highly accessible representation is cognitively much easier and therefore the more likely scenario. Additionally, given that we do observe enhanced retrieval ease for both Cue-Giving and the No-Cue conditions in Experiments 2 and 3, it is clear that the target NP was encoded more robustly compared to the unmodified condition, and given that our participants reported not speaking Korean, the only viable scenario is that the masking characters allowed more time for the encoding of the preceding head noun.

One limitation of this study is the use of masking characters while reading English sentences. Although such a design might be unnatural, and might therefore reduce the ecological validity of the study, we argue that it is sometimes necessary to have such manipulations in order to isolate effects of interest. In fact, previous psycholinguistic research has used similar manipulations such as beeps and construction noise to better understand reference comprehension (Arnold et al., 2007). Moreover, not understanding parts of a sentence is actually quite common under some conditions (e.g., a cocktail party with a lot of ambient noise). Thus, while our design might not be common practice in psycholinguistic research, it is not entirely unnatural.

## Conclusion

We examined the underlying mechanism for the semantic complexity effect (i.e., easier subsequent retrieval of modified relative to unmodified NPs). By replacing modifying English words with masking characters, we increased the time spent maintaining or expecting a head noun thereby heightening attention to the encoding process. The results showed the same degree of facilitation during subsequent retrieval as standard modification, indicating that time spent maintaining or expecting an NP and the concomitant enhanced attention (and not solely or exclusively semantic or syntactic complexity) are the key factors underlying the modification effect.

## Figures and Tables

**Table 1.** Results for Experiment 1. All conditions are compared against the Unmodified condition (red, dashed line in Figure 1).

	that		the		hunters		chased (verb)	
	t	p	t	p	t	p	t	p
Modified	.36	.71	.40	.68	1.06	.28	.32	.74
Cue-Giving	−1.14	.25	−2.83	.005	−1.54	.12	−3.02	.003
No-Cue	.90	.36	−1.08	.27	−.79	.42	−.91	.36
	in		the		*cold*			
	*t*	*p*	t	*p*	*t*	*p*		
Modified	1.33	.18	−.36	.71	−2.97	.003		
Cue-Giving	−2.04	.04	−2.15	.03	−4.58	<.001		
No-Cue	−2.54	.01	−2.07	.03	−3.22	.001		

**Table 2.** Results for Experiment 2. All conditions are compared against the Unmodified condition (red, dashed line in Figure 2).

	that		the		hunters		chased (verb)	
	t	p	t	p	t	p	t	p
Modified	−1.24	.21	.15	.87	2.28	.02	−2.18	.02
Cue-Giving	−2.51	.01	.91	.36	.72	.46	−3.98	<.001
No-Cue	−.54	.58	−.49	.62	−.28	.77	−3.92	<.001
	in		the		*cold*			
	*t*	*p*	*t*	*p*	*t*	*p*		
Modified	−3.47	.001	−3.31	.001	−6.56	<.001		
Cue-Giving	−5.31	<.001	−5.94	<.001	−8.22	<.001		
No-Cue	−5.87	<.001	−3.77	<.001	−7.44	<.001		

**Table 3.** Results for Experiment 3. All conditions are compared against the Unmodified condition (red, dashed line in Figure 3).

	a		trap		when		it (pronoun)	
	t	p	t	p	t	p	t	p
Modified	−2.48	.01	−3.21	.001	−.63	.52	−4.14	<.001
Cue-Giving	−3.22	.001	−3.41	.001	−2.41	.01	−4.13	<.001
No-Cue	−.3.51	<.001	−5.55	<.001	−3.84	<.001	−6.86	<.001
	was		running		*from*			
	*t*	*p*	*t*	*p*	*t*	*p*		
Modified	−1.44	.14	−2.60	.009	−4.49	<.001		
Cue-Giving	−4.18	<.001	−4.98	<.001	−7.21	<.001		
No-Cue	−3.93	<.001	−4.40	<.001	−6.38	<.001		
